# The USTC co-opts an ancient machinery to drive piRNA transcription in *C. elegans*

**DOI:** 10.1101/gad.319293.118

**Published:** 2019-01-01

**Authors:** Chenchun Weng, Joanna Kosalka, Ahmet C. Berkyurek, Przemyslaw Stempor, Xuezhu Feng, Hui Mao, Chenming Zeng, Wen-Jun Li, Yong-Hong Yan, Meng-Qiu Dong, Natalia Rosalía Morero, Cecilia Zuliani, Orsolya Barabas, Julie Ahringer, Shouhong Guang, Eric A. Miska

**Affiliations:** 1 Hefei National Laboratory for Physical Sciences at the Microscale, School of Life Sciences, University of Science and Technology of China, Hefei, Anhui 230027, China;; 2 Wellcome Cancer Research UK Gurdon Institute, University of Cambridge, Cambridge CB2 1QN, United Kingdom; Department of Genetics, University of Cambridge, Cambridge CB2 3EH, United Kingdom;; 3 National Institute of Biological Sciences, Beijing 102206, China;; 4 Structural and Computational Biology Unit, European Molecular Biology Laboratory (EMBL), 69117 Heidelberg, Germany;; 5 Wellcome Sanger Institute, Cambridge CB10 1SA, United Kingdom

**Keywords:** piRNA, PRDE-1, Ruby motif, SNPC, SNPC-4, snRNA, TOFU-4, TOFU-5, U6 RNA

## Abstract

Piwi-interacting RNAs (piRNAs) engage Piwi proteins to suppress transposons and nonself nucleic acids and maintain genome integrity and are essential for fertility in a variety of organisms. In *Caenorhabditis elegans*, most piRNA precursors are transcribed from two genomic clusters that contain thousands of individual piRNA transcription units. While a few genes have been shown to be required for piRNA biogenesis, the mechanism of piRNA transcription remains elusive. Here we used functional proteomics approaches to identify an upstream sequence transcription complex (USTC) that is essential for piRNA biogenesis. The USTC contains piRNA silencing-defective 1 (PRDE-1), SNPC-4, twenty-one-U fouled-up 4 (TOFU-4), and TOFU-5. The USTC forms unique piRNA foci in germline nuclei and coats the piRNA cluster genomic loci. USTC factors associate with the Ruby motif just upstream of type I piRNA genes. USTC factors are also mutually dependent for binding to the piRNA clusters and forming the piRNA foci. Interestingly, USTC components bind differentially to piRNAs in the clusters and other noncoding RNA genes. These results reveal the USTC as a striking example of the repurposing of a general transcription factor complex to aid in genome defense against transposons.

## Abstract (precis)

In this study, Weng et al. provide new insight into the mechanism of piRNA transcription. They used functional proteomics approaches to identify an upstream sequence transcription complex (USTC) that is essential for piRNA biogenesis, and their findings demonstrate the repurposing of a general transcription factor complex, USTC, to aid in genome defense against transposons.

Piwi-interacting RNAs (piRNAs) are a class of small (21- to 30-nucleotide [nt]) RNAs that associate with Piwi proteins, a highly conserved subclass of Argonaute proteins, and play significant roles in fertility and genome stability (Lin and Spradling 1997; Cox et al. 1998; Girard et al. 2006; Carmell et al. 2007; Houwing et al. 2007; Batista et al. 2008; Das et al. 2008; Palakodeti et al. 2008). piRNAs program genome elimination during the sexual reproduction in ciliates (Chalker and Yao 2011) and engage in sex determination, virus defense, regeneration, and neoblast function in animals (Reddien et al. 2005; Palakodeti et al. 2008; Schnettler et al. 2013; Zhao et al. 2013; Kiuchi et al. 2014). piRNAs also play important roles in male fertility in humans (Gou et al. 2017). In *Caenorhabditis elegans*, piRNAs preserve genome integrity in the germline by recognizing and silencing “nonself” genomic loci such as transposons or other foreign nucleic acids and induce chromatin modifications (Malone and Hannon 2009; Ashe et al. 2012; Bagijn et al. 2012; Feng and Guang 2013; Luteijn and Ketting 2013; Mao et al. 2015). In addition to the function of piRNAs in the germline, a recent report provided evidence for the presence of piRNA factors such as PRDE-1 (piRNA silencing-defective 1) and PRG-1 in *C. elegans* neuronal cells (Kim et al. 2018).

*C. elegans* piRNAs are also referred to as 21U-RNAs, given that they are predominantly 21 nt in length and have a strong bias toward 5′ monophosphorylated uracil (Batista et al. 2008; Das et al. 2008; Bagijn et al. 2012; Gu et al. 2012). piRNAs silence transposons and protein-coding genes independently of the endonuclease or slicing activities of the Piwi protein PRG-1. Instead, piRNAs scan for foreign sequences while allowing mismatched pairing with the targeted mRNAs (Ashe et al. 2012; Bagijn et al. 2012; Shirayama et al. 2012). Upon targeting, the piRNA/PRG-1 complex recruits RNA-dependent RNA polymerase (RdRP) to elicit the generation of secondary siRNAs, referred to as “22G-RNAs.” The 22G-RNAs are then loaded onto worm-specific Argonaute proteins (WAGOs) to conduct gene silencing processes (Ashe et al. 2012; Bagijn et al. 2012; Lee et al. 2012; Shirayama et al. 2012; Mao et al. 2015). Meanwhile, “self” mRNAs are protected from piRNA-induced silencing by the CSR-1 Argonaute pathway (Shirayama et al. 2012; Conine et al. 2013; Seth et al. 2013). Therefore, piRNAs are required to initiate the epigenetic silencing, yet the inheritance of the silencing requires 22G-RNAs (Ruby et al. 2006; Batista et al. 2008; Das et al. 2008; Bagijn et al. 2012; Gu et al. 2012; Feng and Guang 2013).

Unlike siRNAs and microRNAs (miRNAs), the biogenesis of piRNAs is a Dicer-independent process (Klattenhoff and Theurkauf 2008; Siomi et al. 2011; Grishok 2013). In *Drosophila*, the long piRNA precursors are transcribed from two different genomic sources, the unistrand and dual-strand clusters, which are further processed and amplified by distinct factors to conduct their respective functions (Brennecke et al. 2007; Saito et al. 2007; Klattenhoff et al. 2009; Handler et al. 2011; Pane et al. 2011; Ipsaro et al. 2012; Goriaux et al. 2014; Mohn et al. 2014; Vourekas et al. 2015; Czech and Hannon 2016). The mouse piRNAs are classified into prepachytene and pachytene piRNAs based on their expression patterns (Aravin et al. 2008; Li et al. 2013)—pachytene piRNA transcription distinctively requiring the transcription factor A-MYB (Li et al. 2013).

piRNAs are expressed in the germline from thousands of genomic loci and mostly from two large genome clusters on chromosome IV. They are first transcribed by RNA polymerase II (Pol II) that initiates precisely 2 nt upstream of the 5′ end of mature piRNAs to generate 25- to 29-nt capped small RNA (csRNA) precursors (Cecere et al. 2012; Gu et al. 2012; Weick et al. 2014). Next, the precursors are decapped at the 5′ end, the first 2 nt are removed, and the extra nucleotides at 3′ ends are trimmed off and methylated to produce mature piRNAs (Billi et al. 2012; Montgomery et al. 2012; de Albuquerque et al. 2014; Weick et al. 2014; Tang et al. 2016). *C. elegans* encodes two Piwi proteins: PRG-1 and PRG-2. Whereas the function of PRG-2 is unknown, mature piRNAs associate with PRG-1 to conduct their functions (Batista et al. 2008; Das et al. 2008; Bagijn et al. 2012). The binding of piRNAs to PRG-1 is important for their production and silencing effect of piRNAs. piRNAs are absent in *prg-1* mutant animals (Wang and Reinke 2008; Gu et al. 2012), and loss of piRNA/PRG-1 complexes leads to reduced fertility (Batista et al. 2008; Das et al. 2008; Wang and Reinke 2008). Interestingly, untrimmed piRNAs with 3′ extensions are stable and associate with PRG-1 yet are unable to robustly recruit other downstream factors and therefore compromise the silencing effect (Tang et al. 2016).

Two types of piRNAs have been described in *C. elegans*. Type I piRNAs are predominantly transcribed from two broad regions on chromosome IV and contain an 8-nt upstream Ruby motif (CTGTTTCA) and a small YRNT motif in which the T corresponds to the first U of the piRNA. Type II piRNAs are present outside of chromosome IV and lack the Ruby motif (Ruby et al. 2006; Gu et al. 2012). Each piRNA is independently transcribed as a short RNA precursor by RNA Pol II. Recent work identified PRDE-1 and SNPC-4 in a complex that binds to the Ruby motif of type I piRNA loci, which are essential for the transcription of piRNA precursors (Kasper et al. 2014; Weick et al. 2014). A Forkhead family transcription factor, *unc-130*, was shown to bind piRNA promoters and has been implicated in piRNA transcription (Cecere et al. 2012). PID-1 may function to promote piRNA processing in the cytoplasm (de Albuquerque et al. 2014). A genome-wide RNAi screen identified seven twenty-one-U fouled-ups (TOFUs) that are engaged in distinct expression and processing steps of piRNAs (Goh et al. 2014). However, it is still unclear how the transcription of piRNA precursors is controlled. Here, we used functional proteomics and identified the upstream sequence transcription complex (USTC) containing PRDE-1, SNPC-4, TOFU-4, and TOFU-5, which bound to the promoters of piRNA precursors to drive their expression in the *C. elegans* germline.

## Results

### Proteomic screens identify a complex containing PRDE-1, SNPC-4, TOFU-4, and TOFU-5 proteins

**Figure 1.**
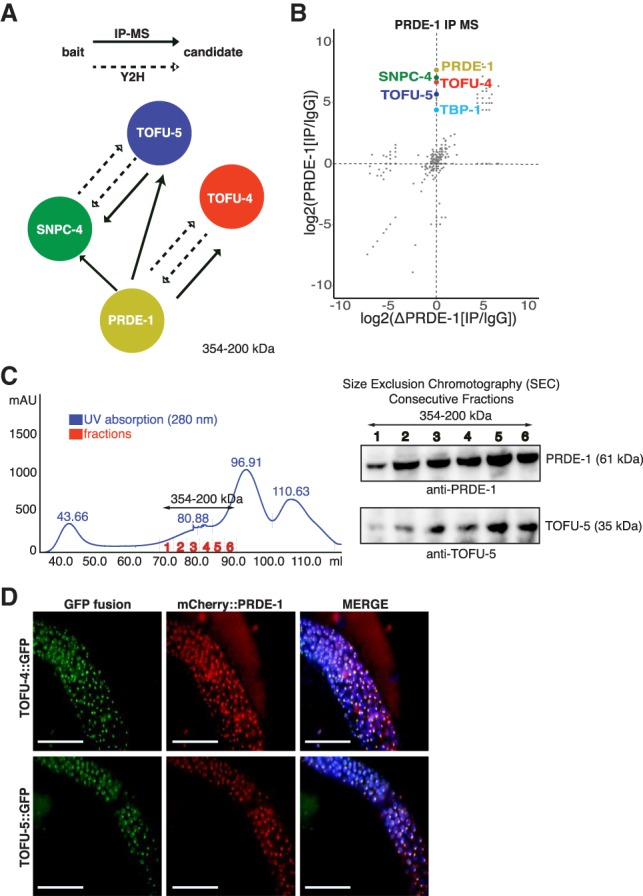
Functional proteomics identify the USTC. (*A*) Summary of protein–protein interaction experiments between PRDE-1, SNPC-4, TOFU-4, and TOFU-5. (*B*) Scatter plot showing the comparison of log_2_ values normalized to IgG of all found proteins in PRDE-1 wild type and *prde-1(mj207)* co-IP–MS. USTC components and TATA-box-binding protein 1 (TBP-1) are displayed. (*C*) Size exclusion chromatography of nuclear extracts followed by Western blot for USTC components PRDE-1 and TOFU-5. Fractions with the highest concentrations of PRDE-1 and TOFU-5 are shown. (*D*) Subcellular colocalization of TOFU-4::GFP and TOFU-5::GFP (green) with mCherry:PRDE-1 (red) in young adult germline nuclei. Bar, 20 µm.

Our previous study identified *prde-1* as being essential for piRNA generation in *C. elegans* (Weick et al. 2014). Later, it was found that a small nuclear RNA (snRNA)-activating complex protein, SNPC-4, colocalize with PRDE-1 and promotes piRNA biogenesis (Kasper et al. 2014). To further understand the transcriptional regulation of piRNAs, we searched for proteins that interact with PRDE-1 using coimmunoprecipitation mass spectrometry (co-IP–MS). Surprisingly, SNPC-4, TOFU-4, and TOFU-5, which were identified in a previous RNAi screen (Goh et al. 2014), were among the top candidates (Fig. 1A,B; Supplemental Fig. S1A; Supplemental Table S1). These proteins significantly separate together from the PRDE-1 knockout immunoprecipitation (Fig. 1B). Thus, we aimed to further investigate whether PRDE-1, SNPC-4, TOFU-4, and TOFU-5 function as a complex in piRNA biogenesis. The physical evidence for the complex was further supported by size exclusion chromatography of *C. elegans* nuclear extracts followed by Western blot analysis. This showed that PRDE-1 and TOFU-5 were present in fractions ranging between 200 and 354 kDa. This is consistent with the expected size of the USTC (251 kDa), assuming 1:1:1:1 stoichiometry (Fig. 1C). To identify proteins directly binding to PRDE-1, we performed yeast two-hybrid (Y2H) experiments and found TOFU-4. This interaction was confirmed by reciprocal Y2H experiments. Considering the previously observed localization dependence between PRDE-1 and SNPC-4 (Kasper et al. 2014), we wanted to search for proteins interacting with SNPC-4. Interestingly, SNPC-4 was found to interact directly with TOFU-5 in Y2H experiments. This interaction was confirmed by co-IP–MS of TOFU-5 (in animals expressing TOFU-5::GFP) and Y2H experiments using TOFU-5::GFP as a bait (Supplemental Fig. S1B; Supplemental Table S1). Altogether, these results raise strong evidence that PRDE-1, SNPC-4, TOFU-4, and TOFU-5 function as a complex during piRNA transcription.

To understand these protein–protein interactions in detail, we generated single-copy GFP-3xFlag-tagged TOFU-4 and TOFU-5 (TOFU-4::GFP and TOFU-5::GFP, respectively) transgenic strains using the Mos1-mediated single-copy insertion (MosSCI) technology (Frokjaer-Jensen et al. 2008). The TOFU-4::GFP and TOFU-5::GFP transgenes rescued the *tofu-4* and *tofu-5* mutant phenotypes, respectively, advocating that the tagged proteins could recapitulate the functions of endogenous proteins (Supplemental Fig. S1C,D). Both TOFU-4::GFP and TOFU-5::GFP exhibit distinct foci in the germline nuclei (Supplemental Fig. S2A,B). Previous work showed that PRDE-1 colocalizes with SNPC-4 and forms distinct foci in germline cell nuclei (Kasper et al. 2014; Weick et al. 2014). We immunostained PRDE-1 with anti-PRDE-1 antibody in TOFU-5::GFP animals and found that PRDE-1 and TOFU-5 also colocalize with each other in the germline nuclei (Supplemental Figs. S1E, S2C). To validate that PRDE-1, TOFU-4, and TOFU-5 function as a complex, we crossed the mCherry::PRDE-1 transgene into TOFU-4::GFP- and TOFU-5::GFP-expressing animals (Fig. 1D). Consistently, both TOFU-4 and TOFU-5 are colocalized with PRDE-1 as distinct foci in germ cell nuclei. Importantly, nuclei in the mitotic zone exhibited two foci, and nuclei in the meiotic zone exhibited one focus, consistent with the ploidy of the cells (Supplemental Fig. S1E; Kasper et al. 2014). Therefore, we conclude that PRDE-1, SNPC-4, TOFU-4, and TOFU-5 likely function as a protein complex to engage in piRNA biogenesis.

### PRDE-1, SNPC-4, TOFU-4, and TOFU-5 are enriched at piRNA clusters and coat the Ruby motif

**Figure 2.**
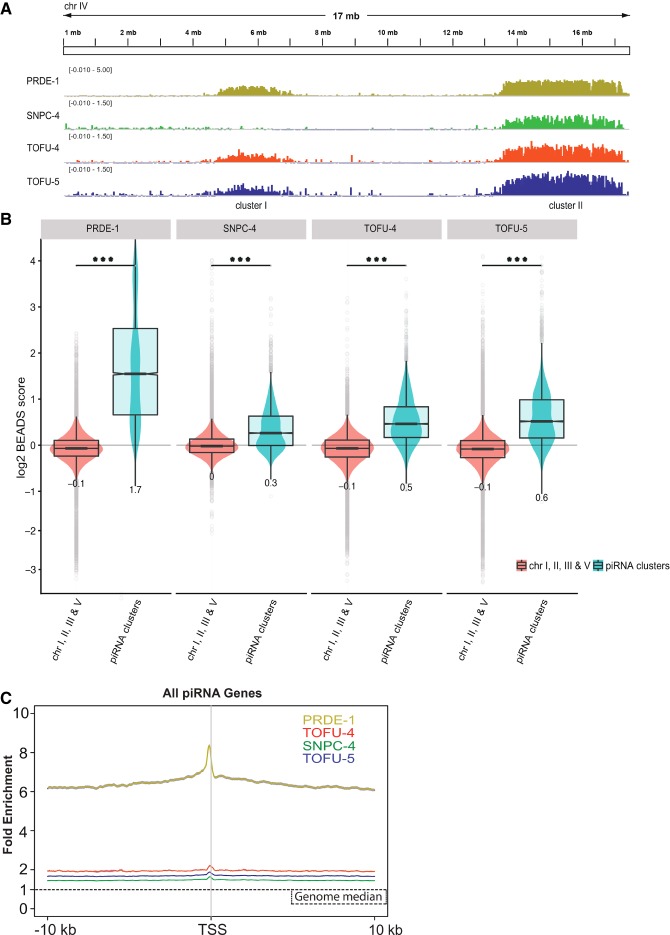
USTC factors coat the piRNA clusters on chromosome IV. (*A*) The binding profiles of USTC factors across chromosome IV. ChIP signal was normalized with rBEADS (bias elimination algorithm for deep sequencing) and log_2_ transformed (PRDE-1 was scaled differently). (*B*) Quantification of ChIP-seq signal on chromosome IV piRNA clusters and other somatic chromosomes (I, II, III, and V). The signal was calculated in 1-kb bins. All USTC factors are significantly enriched on piRNA clusters. (*C*) Enrichment profile of USTC factors around the TSSs of all piRNA genes. The plot is anchored on the first U nucleotide of each piRNA.

SNPC-4 has been shown previously to associate with the chromosome IV piRNA clusters, and the binding depends on the presence of PRDE-1 (Kasper et al. 2014). To test whether PRDE-1, TOFU-4, and TOFU-5 colocalize with SNPC-4 at piRNA clusters, we performed ChIP-seq (chromatin immunoprecipitation [ChIP] combined with high-throughput sequencing) experiments with all four factors in young adults, when piRNA expression is at its peak (Batista et al. 2008; Das et al. 2008); all experiments were done in duplicates and normalized to merged input libraries (Supplemental Fig. S3). We found that all four factors have similar genome-wide binding profiles, with strong enrichment on chromosome IV piRNA clusters (Fig. 2A,B; Supplemental Fig. S4A,B), although SNPC-4 did not appear enriched on the smaller piRNA cluster (cluster I). However, this might be a reflection of lower signal to noise ratio of the SNPC-4 ChIP-seq experiments (Fig. 2A). Finally, PRDE-1, SNPC-4, TOFU-4, and TOFU-5 all “coated” piRNA genes broadly, showing signal around piRNA genes above the genome average and a peak upstream of the transcription start site (TSS) (Fig. 2C).

**Figure 3.**
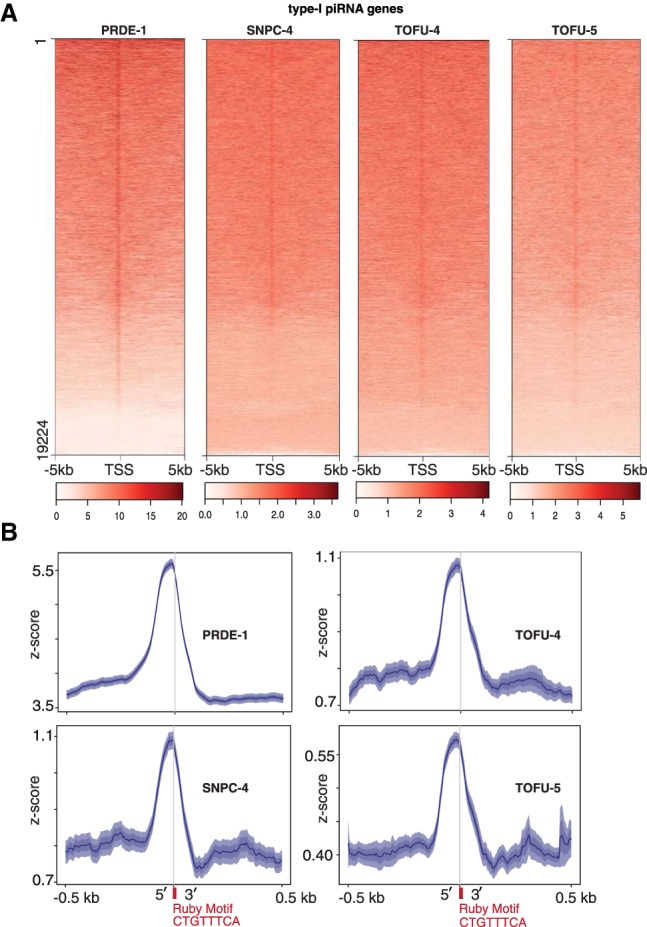
USTC factors are enriched at the Ruby motif upstream of type I piRNA genes. (*A*) Heat map of ChIP-seq binding profiles of USTC factors around type I piRNA TSSs. The BEADS normalization score is plotted 5 kb upstream of and downstream from the first U base of piRNAs. (*B*) Enrichment profile of USTC factors around the Ruby motif upstream of type I piRNA genes within a 500-base-pair (bp) window.

*C. elegans* piRNAs are categorized into type I and type II piRNAs. Type I piRNAs feature the Ruby motif upstream of the TSS of each piRNA transcription unit and are found mostly in the two chromosome IV piRNA clusters, whereas type II piRNAs are more distributed and lack the Ruby motif (Ruby et al. 2006; Gu et al. 2012). To examine whether PRDE-1, SNPC-4, TOFU-4, and TOFU-5 exhibit a preference toward a class of piRNA genes, we plotted heat map profiles of the USTC components around type I and type II piRNA TSSs, respectively (Fig. 3A; Supplemental Fig. S5A). Consistent with our previous finding that PRDE-1 is not required for type II piRNA transcription (Weick et al. 2014), little enrichment was found at these sites (Supplemental Fig. S5A). However, SNPC-4 and TOFU-5 were enriched at type II piRNA genes (Supplemental Fig. S5A). We observed that all four factors exhibit robust enrichment around type I piRNA genes (Fig. 3A) and that Z scores overlapped the Ruby motif (Fig. 3B). Combining the proteomic experiments, subcellular colocalization, and enrichment at type I piRNA genes, we conclude that PRDE-1, SNPC-4, TOFU-4, and TOFU-5 function as a protein complex to promote the biogenesis of piRNAs by binding to the upstream sequence of piRNA genes. We therefore named this complex the USTC.

### The binding of TOFU-5 to the piRNA clusters depends on the other USTC components

A previous study found that the concentration of SNPC-4 at piRNA loci depends on PRDE-1 (Kasper et al. 2014). To investigate the genetic requirements of distinct USTC components for the binding of the piRNA loci, we first examined whether the localization of TOFU-5 to the piRNA loci depends on the presence of other USTC factors.

**Figure 4.**
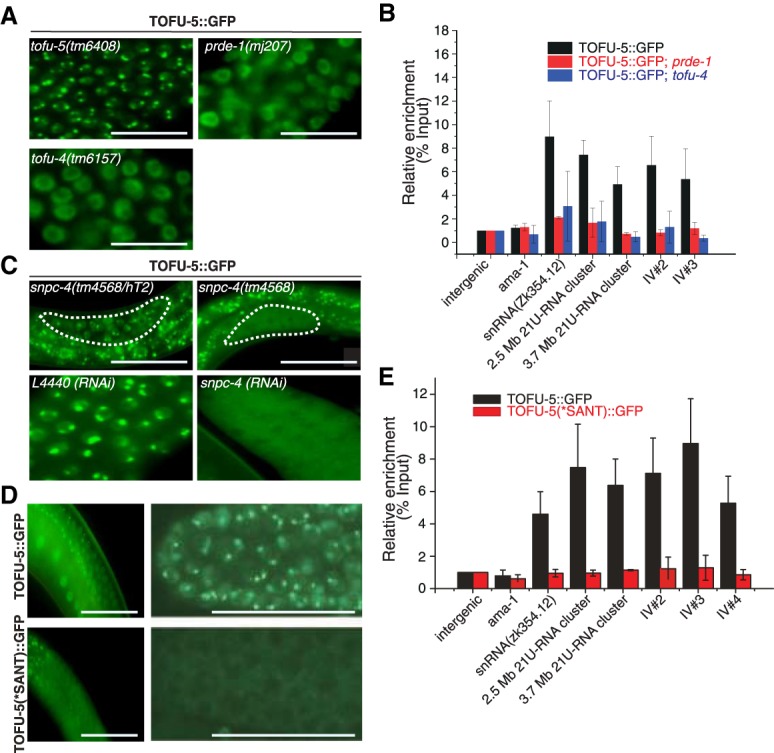
Genetic requirements of TOFU-5 binding to piRNA clusters. (*A*) Images of germline nuclei of young adult animals expressing TOFU-5::GFP. Bar, 20 µm. (*B*) Relative enrichment of TOFU-5 by ChIP assay with an anti-GFP antibody in the indicated young adult animals. *n* = 3 ± SD. Images of germline nuclei of the indicated animals. Primer sets are listed in Supplemental Table S4. (*C*) TOFU-5::GFP failed to localize to nuclei in *snpc-4(−)* young adult animals or upon RNAi feeding against SNPC-4. Bar, 20 µm. (*D*) Images of the indicated germline nuclei of young adult animals. TOFU-5(*SANT)::GFP failed to localize to nuclei in the distal germline. Bar, 20 µm. (*E*) Relative enrichment of TOFU-5::GFP and TOFU-5(*SANT)::GFP by ChIP assay with an anti-GFP antibody in young adult animals. *n* = 3 ± SD.

We crossed the TOFU-5::GFP strain to *tofu-4(tm6157)* and *prde-1(mj207)* mutants and found that TOFU-5 failed to form the subnuclear foci in germline cells in young adult animals (Fig. 4A). Additionally, ChIP-qPCR (ChIP combined with quantitative PCR) of TOFU-5 indicated that TOFU-5 does not bind to piRNA clusters in the absence of PRDE-1 or TOFU-4 (Fig. 4B). SNPC-4 is an essential gene required for the development of *C. elegans*. *snpc-4* mutant animals are embryonic- or larval-lethal (Kasper et al. 2014). We therefore crossed TOFU-5::GFP animals with *snpc-4(tm4568/hT2)* balanced mutant animals and found that TOFU-5 fails to form the subnuclear foci in *snpc-4(tm4568)* homozygous mutant young adult offspring of these animals (Fig. 4C). Remarkably, in *snpc-4(tm4568)* mutants, TOFU-5::GFP accumulates in the germline syncytium, further confirmed by knocking down *snpc-4* by feeding RNAi (Fig. 4C). Both SNPC-4 and TOFU-5 contain a conserved SANT (Swi3 [switching-defective protein 3], Ada2 [adaptor 2], N-CoR [nuclear receptor corepressor], and TFIIIB [transcription factor IIIB]) domain, which may bind to DNA sequences (Boyer et al. 2004). We constructed a TOFU-5(*SANT)::GFP transgenic animal by deleting the SANT domain (Supplemental Fig. S5B). Unlike wild-type TOFU-5, the TOFU-5(*SANT)::GFP fails to form the piRNA foci and is instead enriched in the germline syncytium (Fig. 4D). Consistently, TOFU-5(*SANT)::GFP fails to bind to piRNA clusters, as shown by ChIP assay followed by real-time PCR (Fig. 4E). The similar cytoplasmic locations of TOFU-5(*SANT)::GFP and wild-type TOFU-5 in the absence of SNPC-4 suggest that the SANT domain of TOFU-5 might be required for interaction between SNPC-4 and TOFU-5. In addition, Y2H experiments indicated that the interaction between SNPC-4 and TOFU-5 required the amino acid residues between 768 and 858 of SNPC-4, in which the SANT domain is located. Together, we concluded that the ability of TOFU-5 to bind piRNA clusters and form the piRNA foci depends on other components of the USTC.

### The binding of TOFU-4 to piRNA clusters depends on other USTC factors

**Figure 5.**
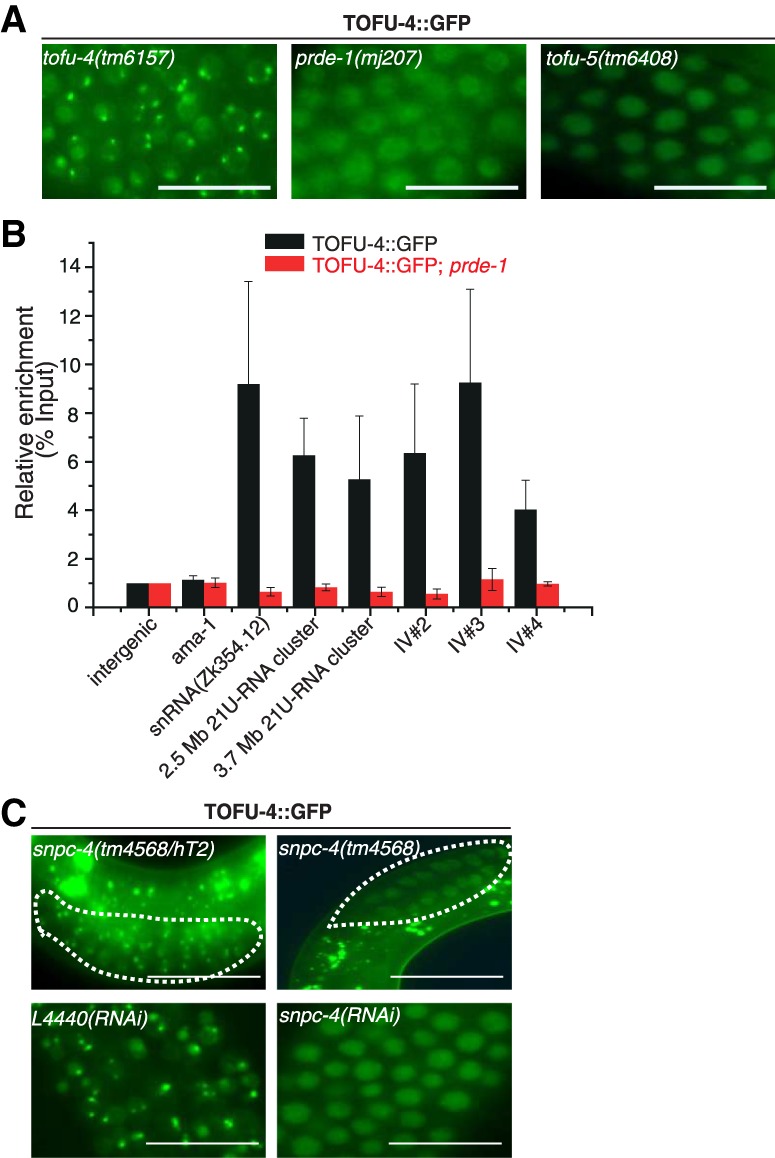
Genetic requirement of TOFU-4 binding to piRNA clusters. (*A*) Images of the indicated germline nuclei of young adult animals expressing TOFU-4::GFP. Bar, 20 µm. (*B*) Relative enrichment of TOFU-4 by ChIP assay with an anti-GFP antibody. *n* = 3 ± SD. Primer sets are listed in Supplemental Table S4. (*C*) TOFU-4::GFP fails to form nuclear foci in the germline in *snpc-4(−)* young adult animals or upon RNAi feeding against SNPC-4.

Next, we examined whether the binding of TOFU-4 to the piRNA loci depends on the presence of other USTC components. We crossed the TOFU-4::GFP transgene into *prde-1(mj207)* mutants and found that PRDE-1 was required for the formation of TOFU-4 piRNA foci (Fig. 5A) and the binding to piRNA clusters (Fig. 5B). We introduced TOFU-4::GFP into *snpc-4(tm4568/hT2)* and *tofu-5(tm6408/hT2)* and found that TOFU-4 fails to form piRNA foci in *snpc-4(tm4586)* and *tofu-5(tm6408)* homozygous mutants, which was further confirmed by knocking down *snpc-4* by feeding RNAi (Fig. 5C). Therefore, we conclude that, similar to our observation for TOFU-5-codependent localization, the binding of TOFU-4 to piRNA clusters depends on the presence of other USTC factors.

### The USTC binds to additional sets of noncoding genes

**Figure 6.**
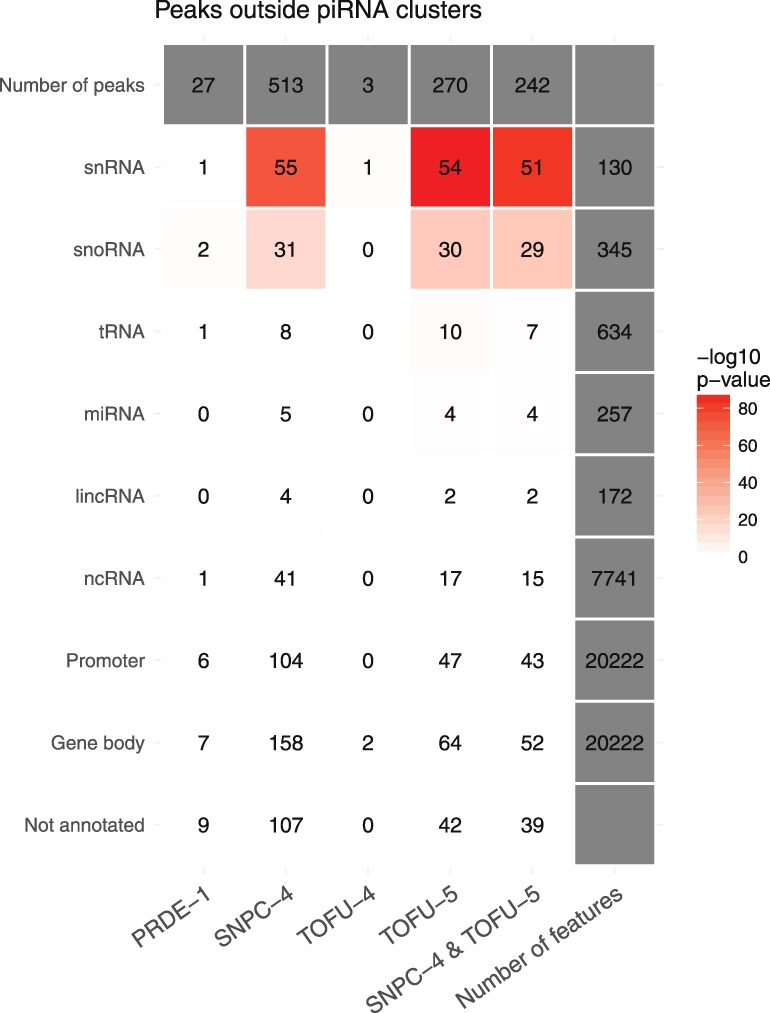
Binding sites of USTC factors outside the piRNA clusters. Gene types of USTC factor-binding sites were analyzed with selected annotations from Ensembl 92. The heat map shows high-confidence USTC factor-binding sites overlapping with selected annotations from Ensembl 92. The bindings sites were detected using overlaps between peak calls on individual replicates. The numbers denote direct overlaps between the peaks and annotated genomic loci, and colors identify the estimation of overlap significance (−log_10_ transformed *P*-value of hypergeometric test for overrepresentation).

**Figure 7.**
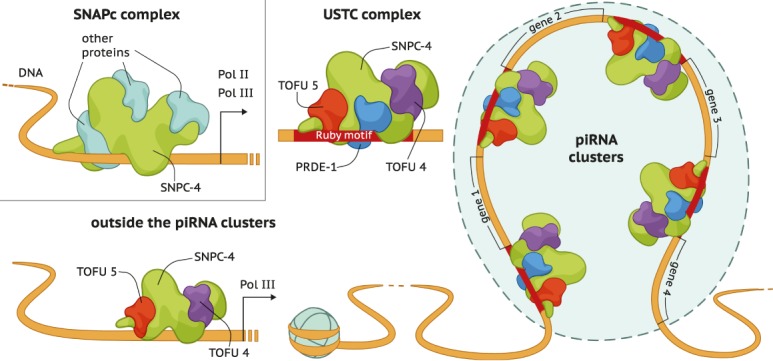
A model for the role of the USTC inside and outside piRNA clusters. Proposed model for the mechanism of the USTC. SNPC-4, TOFU-4, and TOFU-5 bind to ncRNAs outside the piRNA clusters. Together with PRDE-1, all four factors of the USTC engage in piRNA biogenesis.

In addition to the piRNA clusters, SNPC-4 binds canonical SNAPc targets, including RNA Pol II and RNA Pol III transcribed noncoding RNA (ncRNA) genes (Kasper et al. 2014). We therefore investigated whether PRDE-1, SNPC-4, TOFU-4, and TOFU-5 also bound together to other regions in the genome by examining peaks for each factor outside of the piRNA clusters. We found little evidence for PRDE-1 binding outside the piRNA clusters (Fig. 6). However, both SNPC-4 and TOFU-5 were found to bind to a number of snRNA and small nucleolar RNA (snoRNA) genes. As for TOFU-4, we did not observe many peaks, but this might be a reflection of the signal to noise ratio of the TOFU-4 ChIP-seq experiment. Interestingly, SNPC-4 and TOFU-5 are also enriched on specific classes of transposable elements (Supplemental Fig. S6). These results suggest that, in addition to their ancestral functions, including ncRNA transcription, USTC components might have acquired other functions, such as promoting piRNA biogenesis (Fig. 7).

### Identification of TOFU-3 and TATA-box-binding protein 1 (TBP-1) as additional factors required for TOFU-5 binding to piRNA clusters

To further understand the function of the USTC in promoting piRNA transcription, we searched for factors that are required for the formation of TOFU-5 subnuclear piRNA foci. We selected a number of candidate genes from our proteomic experiments and previous genome-wide RNAi screens and carried out a focused candidate RNAi screen for TOFU-5 foci (Supplemental Table S2; Cecere et al. 2012; Goh et al. 2014). Interestingly, we found that both TOFU-3, a candidate from the genome-wide RNAi screen, and TBP-1, a protein that we identified through proteomics, are required for the formation of TOFU-5 piRNA foci (Supplemental Fig. S7A,B; Supplemental Table S2). However, we did not identify any forkhead transcription factors in this screen, which had been shown previously to recognize the Ruby motif (Supplemental Table S2).

## Discussion

Here, by a series of proteomics, imaging, and ChIP-seq experiments, we demonstrate that the four proteins PRDE-1, SNPC-4, TOFU-4, and TOFU-5 function as a complex and bind to the promoter sequences of individual piRNA transcription units. This complex localizes to subnuclear foci and exhibits concentrated binding across the two piRNA-rich domains on chromosome IV. We found that the two categories of piRNAs, classified by the presence of the Ruby motif in their promoter region, may use different combinations of the USTC factors for the promoter recognition. While all of the USTC factors bind to the promoters of piRNA genes, SNPC-4 and TOFU-5 are also enriched on other classes of ncRNA (Figs. 6,7).

### Using functional proteomic methods to identify the USTC

Previously, forward genetic screens have identified PRDE-1 and PID-1 as required factors for piRNA biogenesis in *C. elegans* (de Albuquerque et al. 2014; Weick et al. 2014). Using biochemical approaches, a Forkhead family transcription factor, *unc-130*, was shown to bind piRNA promoters (Cecere et al. 2012). Additionally, a genome-wide RNAi screening identified TOFU genes that are engaged in expression and distinct processing steps of piRNAs (Goh et al. 2014). Here, we combined a series of functional proteomic methods and characterized a USTC that contains PRDE-1, SNPC-4, TOFU-4, and TOFU-5. To our knowledge, this is the first complex involved in piRNA biogenesis in *C. elegans* that has been found. These proteins can interact with each other in a codependent manner and bind to the upstream promoter region of the piRNA transcription units. We further used genome-wide analyses and cell biology approaches and demonstrated a mutual dependency of the components of the USTC in their ability to form the piRNA foci in the germline. Strikingly, one of the USTC factors, SNPC-4, was reported previously as a part of an ancient complex for ncRNA transcription with RNA Pol II and Pol III (Kasper et al. 2014). In summary, we discovered a unique complex that differentially enriches over type I and type II piRNA genes and might engage the transcriptional machinery.

Interestingly, UNC-130, a member of the forkhead transcription factor family, has been reported previously to bind piRNA gene promoters in vitro (Cecere et al. 2012). However, we did not observe UNC-130 or any other forkhead transcription factors’ enrichment with our mass spectrometry approaches using physiological conditions. This might suggest alternative mechanisms that are potentially involved in piRNA biogenesis. It will be interesting to understand the detailed role of forkhead transcription factors using biochemical and proteomic approaches in the future.

### The recognition of piRNA transcription units

*C. elegans* piRNAs are classified into two types. Type I piRNAs are predominantly transcribed from two broad regions from chromosome IV, with an 8-nt upstream Ruby motif (CTGTTTCA) and a small YRNT motif. Type II piRNAs lack the Ruby motif and are present outside of chromosome IV (Ruby et al. 2006; Gu et al. 2012). It has been shown previously that PRDE-1 is required for type I piRNA biogenesis (Weick et al. 2014). The forkhead transcription factors were reported to recognize the Ruby motif as well (Cecere et al. 2012). Here, we showed that, while the four factors of USTC bind type I piRNA promoters, PRDE-1 and TOFU-4 exhibit less binding activity to type II piRNA promoters. Additionally, SNPC-4 and TOFU-5 exhibit binding activity toward type II piRNA promoters. Whether this distinct pattern reflects Ruby-like sequences in type II piRNA promoters that exhibit weaker binding affinity or whether other protein factors are involved in the promoter recognition requires further investigation.

Remarkably, while SNPC-4 and TOFU-5 bind to overlapping sets of non-piRNA promoters, PRDE-1 lacks such binding sites. Therefore, the USTC may play a central role in defining piRNA transcription units and separate piRNA precursors from pre-mRNAs for downstream processing and maturation. RNA Pol II transcribes protein-coding mRNA and also a variety of shorter ncRNAs—most notably spliceosomal U1 and U2 snRNAs (Lykke-Andersen and Jensen 2007; Egloff et al. 2008). Although both piRNA precursors and pre-mRNAs are transcribed by RNA Pol II, the USTC may direct distinct transcription and processing machineries to piRNA units. It will be very interesting to examine the coupling between transcriptional regulation and processing and 3′ end trimming in the biogenesis of piRNAs. Experiments investigating the binding pattern of each component in the absence of other USTC factors will further enlighten our understanding of their mutual regulatory relationships.

### TBP-1 and piRNA biogenesis

*tbp-1* encodes the *C. elegans* ortholog of the human TBP, which plays important roles in transcriptional regulation. TBP-1 has been shown to provide TFIID-like basal transcription activity in human and *C. elegans* extracts, bind specifically to a TATA-box sequence, and interact with TFIIA and TFIIB transcription factors. *tbp-1* activity is required for embryonic and larval development as well as for normal rates of post-embryonic growth. In *Drosophila*, Moonshiner also drives the transcription of piRNA clusters by recruiting TBP-related factor TRF-2 (Andersen et al. 2017).

The function of *tbp-1* in piRNA biogenesis is not yet known. Here, we showed that although TBP-1 did not accumulate in the piRNA foci, it was required for the formation of the piRNA foci and was found to interact with PRDE-1 (Fig. 1B; Supplemental Fig. S7A,B). We speculate that *tbp-1* and the Ruby motif may be required together for USTC binding to piRNA promoters. Here, TBP-1 may act as a bridge to bend the DNA so that the USTC comes closer to the piRNA promoter sequences. Further investigation of the roles of TBP-1 in piRNA biogenesis will shed light on the specific mechanistic understanding of the transcription regulation of piRNAs.

### Chromosome modification in piRNA transcription

Both SNPC-4 and TOFU-5 contain SANT domains (Boyer et al. 2004). Sequence analysis of the SANT domain indicates a strong similarity to the DNA-binding domain (DBD) of Myb-related proteins. SANT domains have been shown to couple histone tail binding to enzymatic activity, including histone acetylation and deacetylation and ATP-dependent chromatin remodeling. Small deletions in the SANT domains may lead to a complete loss of function of the proteins. Here, we show that the deletion of the SANT domain of TOFU-5 relocalizes TOFU-5 from the nucleus to the cytoplasm, disables its ability to bind to piRNA promoters, and alters the piRNA foci. The SANT domain-containing proteins can influence chromatin state, suggesting that SNPC-4 and TOFU-5 can modulate nucleosome organization and/or histone modifications in the piRNA regions. Consistently, the piRNA regions exhibited decreased nucleosome density in young adult animals (Cecere et al. 2012). It is still unclear whether the presence of the SANT domain is required for the cytoplasm-to-nucleus import of TOFU-5.

Chromatin modifications play important roles in small RNA biogenesis. In *Drosophila*, transcription of piRNA clusters is enforced through RNA Pol II preinitiation complex formation within repressive heterochromatin, in which the heterochromatin protein-1 variant Rhino recruits Moonshiner (Andersen et al. 2017). In *C. elegans*, two piRNA clusters on chromosome IV are nucleosome-depleted regions (Cecere et al. 2012). In our PRDE-1 Y2H experiment, *set-6* (a gene encoding a putative H3K9 methyltransferase) and *set-16* (a gene encoding a putative H3K4 methyltransferase) were identified. However, these two proteins and other chromatin modification factors were not required for the formation of piRNA foci (Supplemental Table S1). Therefore, we speculate that either the biogenesis of piRNAs is independent of chromatin modification processes or certain chromatin factors act together to promote the formation of piRNA foci. Further analysis of the properties of chromatin, including histone modifications, in the germline will be necessary to directly explore the relationship between the chromatin state, the USTC, and piRNA expression.

## Materials and methods

### Strains

Bristol strain N2 was used as the standard wild-type strain. All strains were grown at 20°C unless specified. The strains used in this study are listed in Supplemental Table S3.

### Antibody production

Antibodies against PRDE-1 and TOFU-5 were produced at the Laboratory Animal Resources (European Molecular Biology Laboratory, Heidelberg). For this, New Zealand White female rabbits were immunized with the recombinant full-length proteins together with complete Freund's adjuvant. After the priming immunization, four further booster immunizations were performed every 3 wk. The harvested serum was purified by affinity purification using the recombinant full-length PRDE-1 or TOFU-5, respectively.

### Immunoprecipitation and MS analysis

Nonsynchronized transgenic animals expressing TOFU-5::GFP or TOFU-5(*SANT)::GFP were resuspended in an equal volume of 2× lysis buffer (50 mM Tris-HCl at pH 8.0, 300 mM NaCl, 10% glycerol, 1% Triton X-100, Roche Complete EDTA-free protease inhibitor cocktail, 10 mM NaF, 2 mM Na_3_VO_4_) and lysed in a FastPrep-24 5G homogenizer. The supernatant of lysate was incubated with homemade anti-GFP beads for 1 h at 4°C. The beads were then washed three times with cold lysis buffer. Proteins were eluted with chilled elution buffer (100 mM glycine-HCl at pH 2.5). Eluates were precipitated with TCA or cold acetone and dissolved in 100 mM Tris (pH 8.5) and 8 M urea. The proteins were reduced with TCEP, alkylated with IAA, and finally digested with trypsin overnight at 37°C. The liquid chromatography-tandem MS (LC-MS/MS) analysis of the resulting peptides and the MS data processing approaches were conducted as described previously (Feng et al. 2017). Samples were analyzed on an LTQ Orbitrap Velos Pro (Thermo Fisher Scientific) LC/MS-MS system. Data were searched with Mascot (version 2.2.03; Matrix Science) using the UniProt *C. elegans* database, and unique peptides were analyzed with Scaffold 3. MS data normalization for scatter and MA plots was carried out with a custom R script. All raw data and normalized MS data are shown in Supplemental Tables S5–S8.

The wild-type strain was var. Bristol N2 (Brenner 1974), and the SX2499 (mj207) strain was a *prde-1* mutant. Synchronized nematodes were collected at young adult stage in PBS with EDTA-free Complete protease inhibitor cocktail tablets (Roche) and frozen in liquid nitrogen. Qproteome cell compartment kit (Qiagen) was used to purify nuclear fractions that were diluted with PBSCM buffer (1× PBS, 1 mM CaCl_2_, 1 mM MgCl_2,_ 1× EDTA-free Complete protease inhibitor cocktail). Protein concentration was measured with BCA protein measurement kit (Pierce). Anti-PRDE-1 antibody KSOJ (0.88 mg/mL) was coupled to 5 µg of Dynabeads per 1 mg of beads according to the manufacturer's instructions. Uncoupled IgG beads were used as a control. Beads were incubated with nuclear extracts overnight at 4°C and washed eight times with PBSCM, and proteins were eluted in 80 µL of elution buffer (100 mM TAEB, 4% SDS) by boiling for 30 min at 60°C. The elutes were precipitated in TCA or cold acetone and dissolved in 100 mM Tris (pH 8.5) and 8 M urea. The proteins were reduced with TCEP and alkylated with IAA. The samples were digested with trypsin overnight at 37°C, and each sample was run separately.

### Y2H analysis

The coding sequence of the *C. elegans* TOFU-4 protein fragment (amino acids 1–223), the full-length *C. elegans* TOFU-5 protein (amino acids 1–311), the full-length *C. elegans* PRDE-1 protein (amino acids 1–526), and the full-length *C. elegans* SNPC-4 protein (amino acids 1–928) (NCBI sequence reference NM_060011.4) were PCR-amplified and cloned into pB27 as a C-terminal fusion to LexA-DBD (LexA-TOFU4, LexA-TOFU5, LexA-SNPC-4, and LexA-Prde-1). Constructs were checked by sequencing the entire insert and used as bait to screen a random-primed *C. elegans* mixed-stage cDNA library constructed into pP6. pB27 and pP6 were derived from the original pBTM116 (Vojtek and Hollenberg 1995) and pGADGH plasmids, respectively. Clones (91 million–137 million, depending on the experiment; 10-fold the complexity of the library) were screened using a mating approach with YHGX13 (Y187 *ade2-101::loxP-kanMX-loxP*, *MAT*) and L40:*Gal4* (*MAT*) yeast strains as described previously (Fromont-Racine et al. 1997). Depending on the bait, 148–365 His^+^ colonies were selected on a medium lacking tryptophan, leucine, and histidine or supplemented with 0.5 mM 3-aminotriazole to handle bait autoactivation for LexA-Prde-1. The prey fragments of the positive clones were amplified by PCR and sequenced at their 5′ and 3′ junctions. The resulting sequences were used to identify the corresponding interacting proteins in the GenBank database (NCBI) using a fully automated procedure. A confidence score (PBS [predicted biological score]) was attributed to each interaction as described previously (Formstecher et al. 2005). The PBS scores have been shown to positively correlate with the biological significance of interactions (Rain et al. 2001; Wojcik et al. 2002). Y2H screening was performed with the help of Hybrigenics Services.

### Size exclusion chromatography

Five milliliters of worm nuclear extract, prepared using a Qproteome cell compartment kit (Qiagen), was injected into a Superose6 xK 16/70 (GE Healthcare). One-milliliter fractions (starting after the void volume of 40 mL) were collected, and proteins were precipitated with TCA, separated by gel electrophoresis, and transferred to a nitrocellulose membrane using a semidry transfer system (Bio-Rad).

### Construction of plasmids and transgenic strains

For *TOFU-4::GFP*, a TOFU-4 promoter and CDS region were PCR-amplified with the primers 5′-GCAGATCTTCGAATGCATCGAATTGAAAATGAGAAAAAACTG-3′ and 5′-ATAGCTCCACCTCCACCTCCGAATTCTGCGTCTACCATCA-3′ from N2 genomic DNA. A GFP::3xFlag region was PCR-amplified with the primers 5′-GGAGGTGGAGGTGGAGCTATGAGTAAAGGAGAAGAAC-3′ and 5′-TCACTTGTCATCGTCATCCT-3′ from plasmid pSG085. A TOFU-4 3′ UTR (untranslated region) was PCR-amplified with the primers 5′-AGGATGACGATGACAAGTGAAAGAATTTTTATCCGAGTCAC-3′ and 5′-AGATATCCTGCAGGAATTCCGAAACTTGATTTTCAAAATTTA-3′ from N2 genomic DNA. The ClonExpress MultiS one-step cloning kit (Vazyme, C113-02) was used to insert the *TOFU-4::GFP::3xFlag* fusion gene into the pCFJ151 vector. The transgene was integrated onto the *C. elegans* chromosome II by the MosSCI method (Frokjaer-Jensen et al. 2008).

For *TOFU-5::GFP*, a TOFU-5 promoter and CDS region were PCR-amplified with the primers 5′-GCAGATCTTCGAATGCATCGTTGGTTCAACCTGGAATTAA-3′ and 5′-ATAGCTCCACCTCCACCTCCATTGGACGATGTAGATGGCTG-3′ from N2 genomic DNA. A GFP::3xFlag region was PCR-amplified with the primers 5′-GGAGGTGGAGGTGGAGCTATGAGTAAAGGAGAAGAAC-3′ and 5′-AAAAATTTCACTTGTCATCGTCATCCTTGT-3′ from plasmid pSG085. A TOFU-5 3′ UTR was PCR-amplified with the primers 5′-CGATGACAAGTGAAATTTTTATTAATTTTTTG-3′ and 5′-AGATATCCTGCAGGAATTCCCATGGCTTGGAATTGAAG-3′ from N2 genomic DNA. The ClonExpress MultiS one-step cloning kit (Vazyme, C113-02) was used to insert the *TOFU-5::GFP::3xFlag* fusion gene into the pCFJ151 vector. The transgene was integrated onto the *C. elegans* chromosome II by the MosSCI system.

For TOFU-5(*SANT)::GFP, plasmid was PCR-amplified with the primers 5′-TCGCAGCTTCATCGAAGAGATAAAGTAAGT-3′ and 5′-TCTCTTCGATGAAGCTGCGACCTTCTGCGA-3′ from *TOFU-5::GFP::3xFlag* plasmid. The transgene was integrated onto the *C. elegans* chromosome II by the MosSCI system.

### RNAi

RNAi experiments were conducted as described previously (Timmons et al. 2001). Images were collected using a Leica DM2500 microscope.

### ChIP

ChIP experiments were performed as described previously with hypochlorite-isolated embryos or young adults (Guang et al. 2010). Animals were cross-linked in 2% formaldehyde for 30 min. Fixation was quenched with 0.125 M glycine for 5 min at room temperature. After cross-linking, samples were resuspended in FA buffer (50 mM Tris/HCl at pH 7.5, 1 mM EDTA, 1% Triton X-100, 0.1% sodium deoxycholate, 150 mM NaCl) with a proteinase inhibitor tablet (Roche, 04693116001) and sonicated for 20 cycles at medium output (each cycle: 30 sec on and 30 sec off) with a Bioruptor 200. Lysates were precleared and then immunoprecipitated with 1.5 µL of anti-GFP antibody (Abcam, ab290) for SNPC-4, TOFU-4, and TOFU-5 and 5 µL of anti-PRDE-1 for PRDE-1 overnight at 4°C. Antibody-bound complexes were recovered with Dynabeads Protein A. Following extensive sequential washes with 150, 500, and 1 M NaCl, DNA was treated with RNase (Roche) and ProK (New England Biolabs). Finally, resulting DNA samples were purified with QIAquick PCR purification kit (Qiagen, 28104).

### ChIP-seq

The DNA samples from ChIP experiments were sent to in-house sequencing for library preparation and sequencing. Briefly, 10–300 ng of ChIP DNA was combined with End Repair Mix and incubated for 30 min at 20°C followed by purification with a QIAquick PCR purification kit (Qiagen). The DNA was then incubated with A-tailing mix for 30 min at 37°C. The 3′ end adenylated DNA was incubated with the adapter in the ligation mix for 15 min at 20°C. The adapter-ligated DNA was amplified by several rounds of PCR amplification and purified using a 2% agarose gel to recover the target fragments. The average molecule length was analyzed on the Agilent 2100 bioanalyzer instrument (Agilent DNA 1000 Reagents). The library was quantified by qPCR (TaqMan probe). The libraries were further amplified on cBot to generate the clusters on the flow cell and sequenced with a single-end 50 method on a HiSeq1500 system.

### Alignment to reference genome for ChIP-seq data

Chip-seq libraries were sequenced using Illumina HiSeq. Reads were aligned to the ce11 assembly of the *C. elegans* genome using BWA version 0.7.7 (Li and Durbin 2010) with default settings (BWA-backtrack algorithm). The SAMtools version 0.1.19 “view” utility was used to convert the alignments to BAM format. Normalized ChIP-seq coverage tracks were generated using the BEADS (bias elimination algorithm for deep sequencing) algorithm (Cheung et al. 2011).

### Summed ChIP-seq input and in-house blacklist

We generated summed input BAM files by combining good quality ChIP-seq input experiments from different extracts (eight experiments for formaldehyde and five experiments for EGS extracts). The same summed inputs were used for BEADS normalization and peak calls. We observed that despite using input files for MACS2 (Zhang et al. 2008) and filtering against modENCODE, some regions of high signal in input were still called as peaks. To overcome this problem, we created an in-house blacklist by running MACS2 with default settings and no input mode. The blacklist regions were refined by discarding regions with a MACS2 score <100 and clustering peaks within 500 base pairs (bp). This procedure created 90 new regions in addition to 122 already covered by the modENCODE blacklist.

### Peak calls

ChIP-seq peaks were called using MACS2 version 2.1.1 (Feng et al. 2012) with a permissive 0.05 *q*-value cut-off and a fragment size of 150 bp against summed ChIP-seq input. To generate sharp peak call sets, we obtained peak summits and extended them 150 bp upstream and downstream, creating 300-bp regions around summit calls. Furthermore, we combined ChIP-seq replicates by intersecting these regions and setting the final peak size back to 300 bp. Finally, peaks overlapping nonmappable (GEM mappability <25%) or blacklisted regions were discarded. This produced sharp uniform peaks suitable for further quantitative analyses.

### Peak call annotation

PRDE-1, SNPC-4, TOFU-4, and TOFU-5 peak calls were classified using selected annotations from Ensembl version 92. The peak was assigned to a given class if it directly overlapped with annotated loci. The assignments were exclusive (a peak could be assigned to only a single class), giving the following order of priority: snRNA, snoRNA, transfer RNA (tRNA), miRNA, large intergenic ncRNA (lincRNA), ncRNA, promoter, and gene body. Promoters were defined as 500 bases upstream of annotated TSSs. The overlap significance was estimated using a hypergeometric test for overrepresentation.

### ChIP-seq data aggregation and visualization

SeqPlots (Stempor and Ahringer 2016) software was used to visualize PRDE-1, SNPC-4, TOFU-4, and TOFU-5 ChIP-seq profiles over Ruby motif locus, piRNAs gene, snRNA, and tRNA average as average aggregated plots and heat maps. The Integrative Genomics Viewer genome browser (Robinson et al. 2011) was applied to visualize signals genome-wide and on piRNA clusters.

### ChIP-seq signal quantifications

To quantify differences in PRDE-1, SNPC-4, TOFU-4, and TOFU-5 binding between piRNA clusters and somatic chromosomes, we quantified the BEADS-normalized log_2_-scaled signal in 1-kb bins divided into piRNA clusters and chromosomes I, II, III, and V. The signal was obtained using the bigWigSummary utility from the Kent library (Kent et al. 2010) implemented in rtracklayer package in R. Next, the signal was represented as an overlaid violin plot (showing signal distribution) and Tukey box plot (showing estimation of statistical significance of difference between medians as notches).

### Statistics

Bar graphs with error bars are presented with mean and standard deviation. All of the experiments were conducted with independent *C. elegans* animals for the indicated *N* times. Statistical analysis was performed with two-tailed Student's *t*-test.

### Data availability

All raw and normalized sequencing data have been deposited to Gene Expression Omnibus under submission number GSE112682.

### piRNA gene annotations

piRNA annotations were downloaded from the piRBase online database (http://www.regulatoryrna.org/database/piRNA). Genomic coordinates of piRNA genes were obtained by SAMtools against the *C. elegans* ce10 genome assembly. Type II piRNA genes were obtained from a previous publication (Gu et al. 2012). Type I piRNA gene lists were created by filtering the piRBase annotations with type II piRNA genes.

## Supplementary Material

Supplemental Material
