# Supplementary material for: The USTC co-opts an ancient machinery to drive piRNA transcription in C. elegans
Source: Genes Dev. 2019 Jan 1;33(1-2):90–102. doi: 10.1101/gad.319293.118 (PMC6317315; doi:10.1101/gad.319293.118)
Supplement: Supplemental Material [file supp_33_1-2_90__index.html]

The USTC co-opts an ancient machinery to drive piRNA transcription in C. elegans — Supplemental Material 

# The USTC co-opts an ancient machinery to drive piRNA transcription in *C. elegans*

## Supplemental Material

- SupplementalTableS1.xlsx
- SupplementalTableS8.xlsx
- SupplementalTableS6.csv
- SupplementalTableS4.xlsx
- SupplementalTableS2.xlsx
- SupplementalTableS7.csv
- SupplementalTableS5.csv
- SupplementalTableS3.xlsx
- Supplemental\_Material.pdf
