# Supplementary material for: The USTC co-opts an ancient machinery to drive piRNA transcription in C. elegans
Source: Genes Dev. 2019 Jan 1;33(1-2):90–102. doi: 10.1101/gad.319293.118 (PMC6317315; doi:10.1101/gad.319293.118)
Supplement: Supplemental Material [file supp_gad.319293.118_Supplemental_Material.pdf]

## SUPPLEMENTARY FIGURE LEGENDS

**Figure S1. TOFU-4::GFP and TOFU-5::GFP transgenes are functional.**

(*A*) MA plots of PRDE-1 *wild type* and *prde-1(mj207)* co- IP-MS data normalized to IgG.

(*B*) Scatter plot showing the differences between TOFU-5 wild type and TOFU-5 SANT domain deletion co-IP-MS.

(*C*) TOFU-4 is required for normal fertility, TOFU-4::GFP rescued the progeny defect of *tofu-4(tm6157)* mutant. Average brood size of wild-type, *tofu-4(tm6157)*, *prg-1(n4503)* mutant stains and *tofu-4* rescue lines at 20°C and 25°C.

(*D*) TOFU-5 is required for normal fertility, TOFU-5::GFP rescued the sterile phenotype of *tofu-5(tm6408)* mutant. Average brood size of wild-type and *tofu-5(tm6408)/hT2*, *tofu-5(tm6480)* rescue line, *tofu-5(tm6408)* at 20°C. (n) Number of parental adults used.

(*E*) Zoomed in images of subcellular colocalization of TOFU-4::GFP and TOFU-5::GFP (green) with mCherry:PRDE-1 (red) in young adult germline nuclei. Scale bar, 20 µm.

**Figure S2. Expression pattern of TOFU-4, TOFU-5, and PRDE-**1.

(*A*) Image of TOFU-4 in a dissected gonad of a young adult animal.

(*B*) Image of TOFU-5 in a dissected gonad of a young adult animal.

(*C*) Colocalization of PRDE-1 and TOFU-5. Scale bar, 20 µm.

**Figure S3. Pearson Correlation between ChIP-seq replicates.**

Pearson correlation between the ChIP-seq replicates of USTC factors using 1 kb bins.

**Figure S4. The USTC complex binds piRNA clusters on chromosome IV.**

(*A*) Log2 fold enrichment of ChIP-seq individual replicates from the USTC factors on the piRNA clusters.

(*B*) Log2 BEADS scores of the USTC factors in piRNA cluster I and II of chromosome IV over the genome average.

**Figure S5. Chromatin and DNA binding pattern of USTC components and expression levels of TOFU-5(*SANT).**

(*A*) Fold enrichment of the USTC complex ChIP-seq over type II piRNA genes. The BEADS score was plotted 5 kb upstream and downstream of the 1st U base of piRNAs.

(*B*) Expression pattern of TOFU-5(*SANT) deletion compared to wild type.

**Figure S6. |Association of USTC factors with repeat elements.**

Dfam2.0 repeat annotations were used for the analysis. Elements that showed at least one intersection with the USTC factors are shown.

**Figure S7. Expression pattern of TBP-1.**

(*A*) Genetic requirement for TOFU-5::GFP in *tofu-3* mutant and *tbp-1*(RNAi). Scale bar, 20 μm.

(*B*) Image of TBP-1::GFP in a dissected gonad of a young adult. Scale bar, 20 μm.

Figure S1

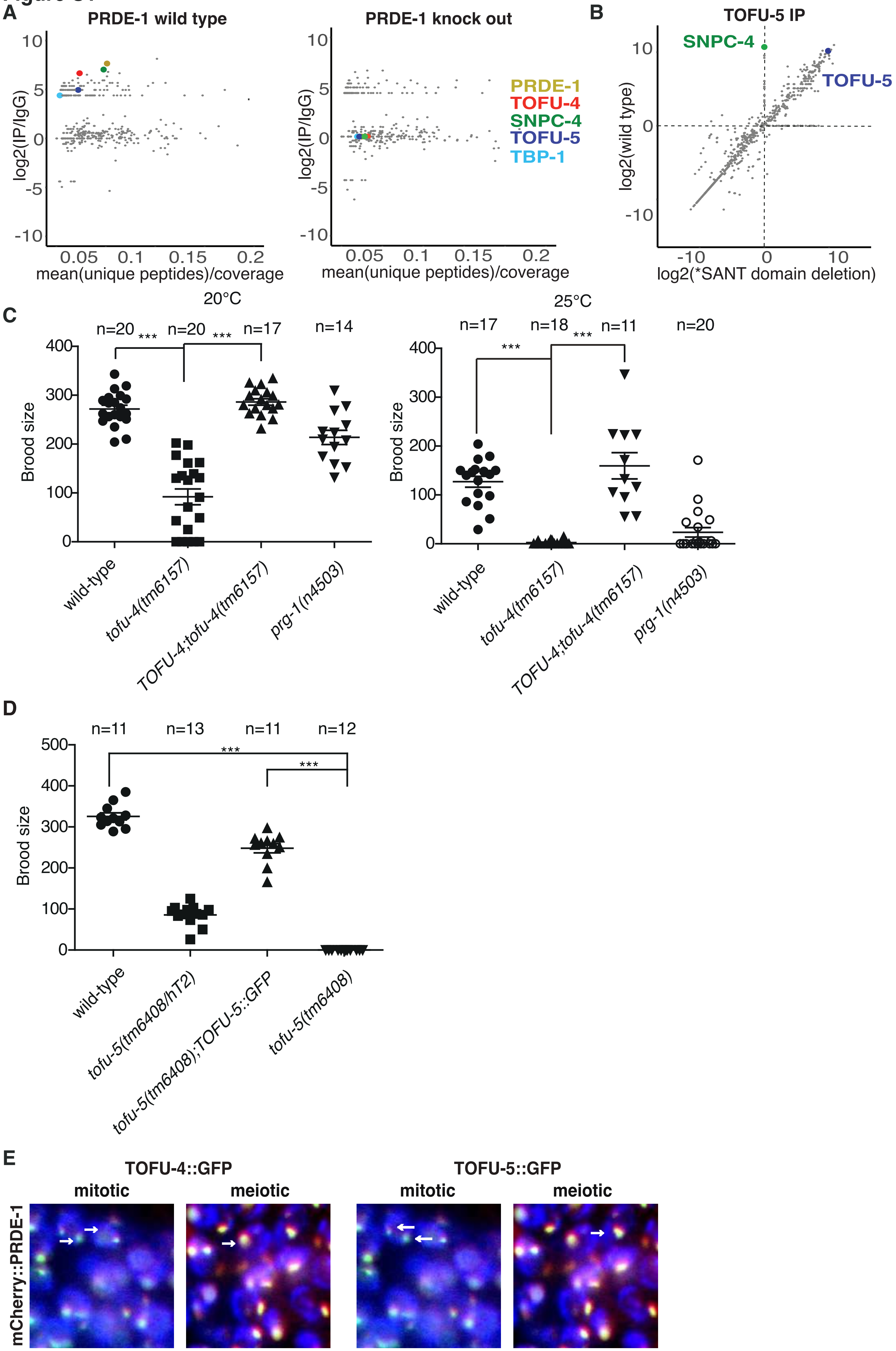
A
PRDE-1 wild type
PRDE-1 knock out
log2(IP/IgG)
mean(unique peptides)/coverage
PRDE-1
TOFU-4
SNPC-4
TOFU-5
TBP-1
B
TOFU-5 IP
SNPC-4
TOFU-5
log2(wild type)
log2(*SANT domain deletion)
C
20°C
25°C
n=20
n=20
n=17
n=14
n=17
n=18
n=11
n=20
***
Brood size
wild-type
tofu-4(tm6157)
TOFU-4;tofu-4(tm6157)
prg-1(n4503)
D
n=11
n=13
n=11
n=12
***
Brood size
wild-type
tofu-5(tm6408/hT2)
tofu-5(tm6408);TOFU-5::GFP
tofu-5(tm6408)
E
TOFU-4::GFP
TOFU-5::GFP
mitotic
meiotic
mCherry::PRDE-1

## Figure S2

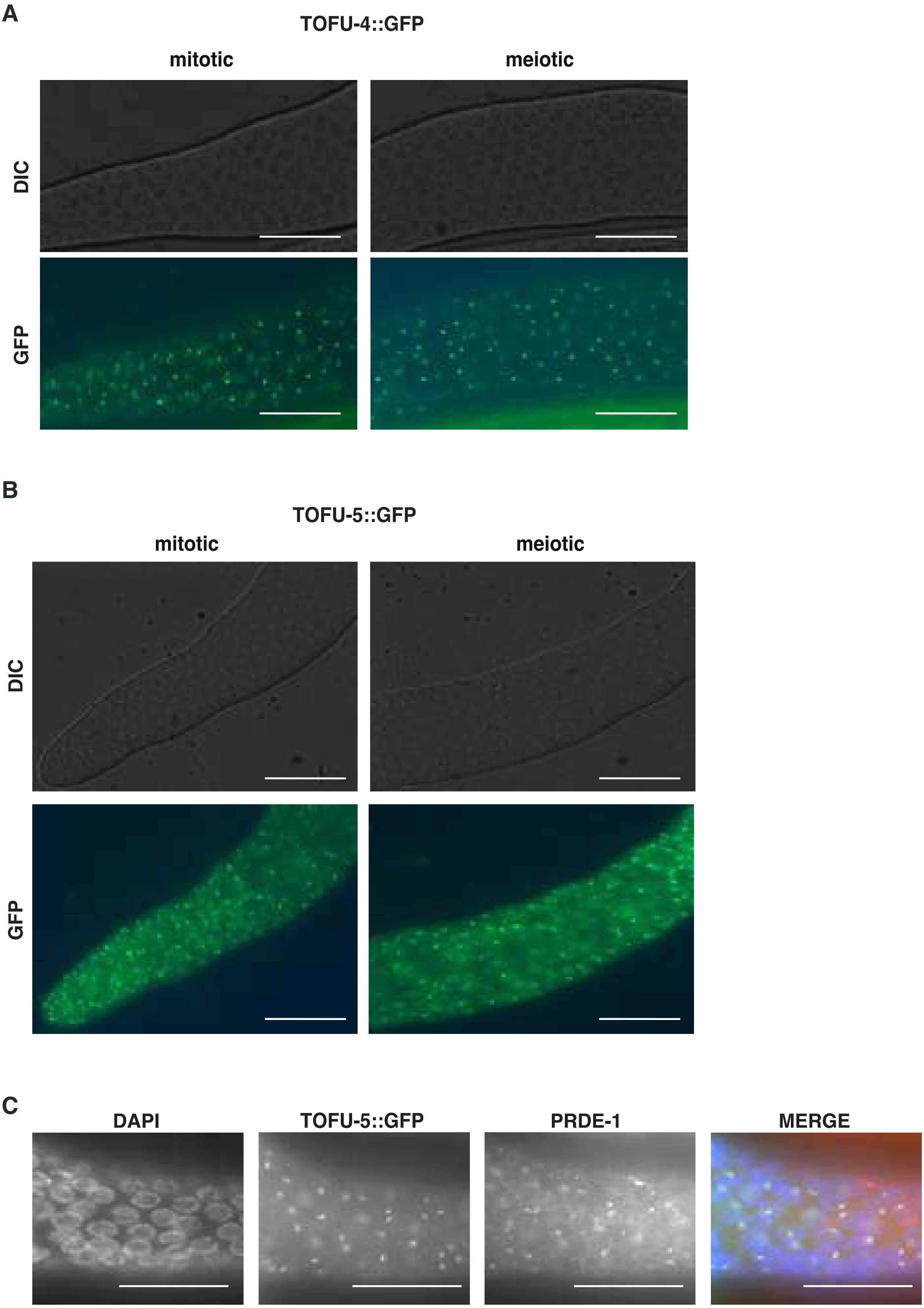
A
TOFU-4::GFP
mitotic
meiotic
DIC
GFP
B
TOFU-5::GFP
mitotic
meiotic
DIC
GFP
C
DAPI
TOFU-5::GFP
PRDE-1
MERGE

# Figure S3

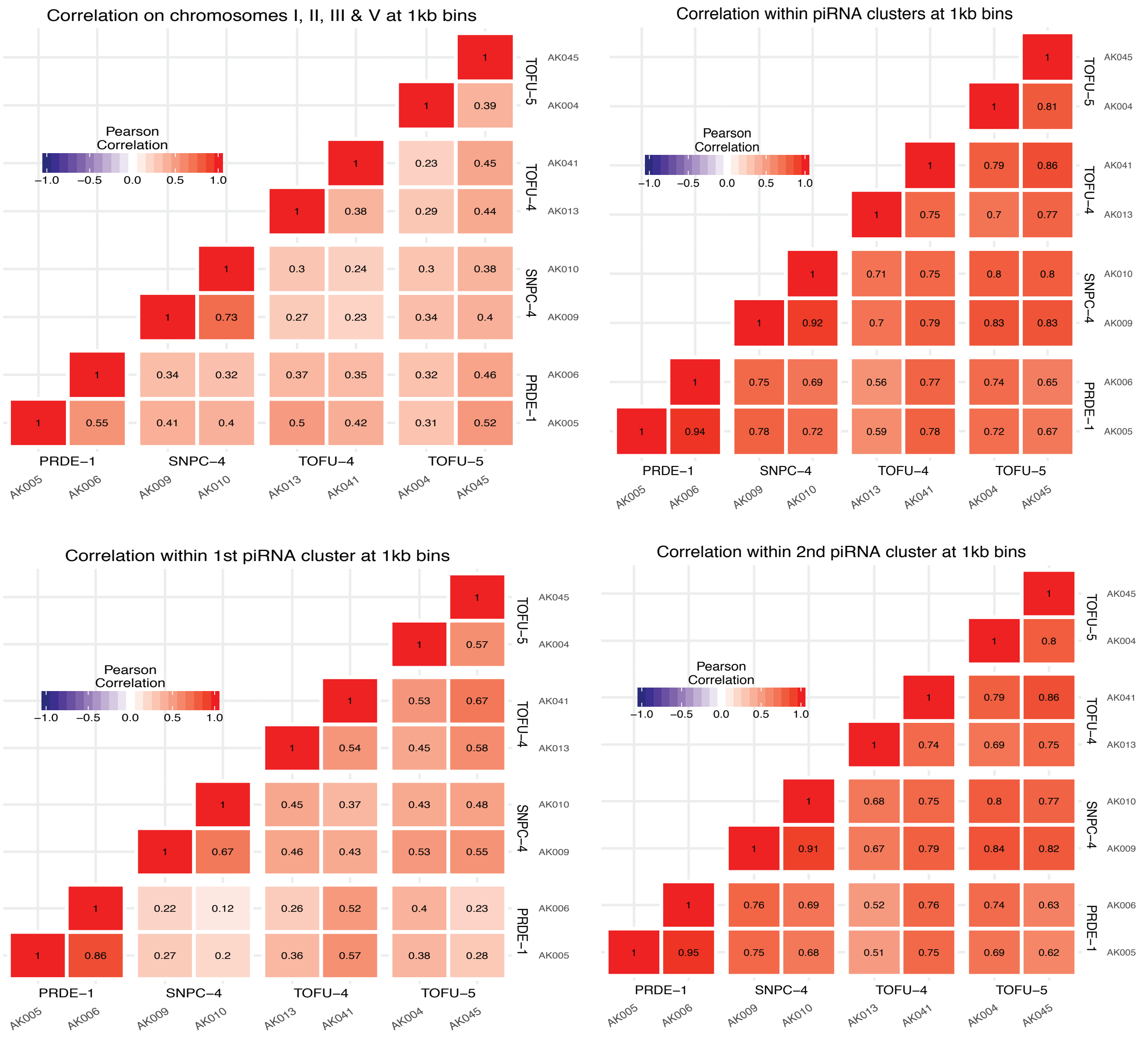
Correlation on chromosomes I, II, III & V at 1kb bins
Pearson Correlation
−1.0 −0.5 0.0 0.5 1.0
1
1 0.39
1 0.23 0.45
1 0.38 0.29 0.44
1 0.3 0.24 0.3 0.38
1 0.73 0.27 0.23 0.34 0.4
1 0.34 0.32 0.37 0.35 0.32 0.46
1 0.55 0.41 0.4 0.5 0.42 0.31 0.52
TOFU−5 TOFU−4 SNPC−4 PRDE−1
AK045 AK004 AK041 AK013 AK010 AK009 AK006 AK005
PRDE−1 SNPC−4 TOFU−4 TOFU−5
AK005 AK006 AK009 AK010 AK013 AK041 AK004 AK045
Correlation within piRNA clusters at 1kb bins
Pearson Correlation
−1.0 −0.5 0.0 0.5 1.0
1
1 0.81
1 0.79 0.86
1 0.75 0.7 0.77
1 0.71 0.75 0.8 0.8
1 0.92 0.7 0.79 0.83 0.83
1 0.75 0.69 0.56 0.77 0.74 0.65
1 0.94 0.78 0.72 0.59 0.78 0.72 0.67
TOFU−5 TOFU−4 SNPC−4 PRDE−1
AK045 AK004 AK041 AK013 AK010 AK009 AK006 AK005
PRDE−1 SNPC−4 TOFU−4 TOFU−5
AK005 AK006 AK009 AK010 AK013 AK041 AK004 AK045
Correlation within 1st piRNA cluster at 1kb bins
Pearson Correlation
−1.0 −0.5 0.0 0.5 1.0
1
1 0.57
1 0.53 0.67
1 0.54 0.45 0.58
1 0.45 0.37 0.43 0.48
1 0.67 0.46 0.43 0.53 0.55
1 0.22 0.12 0.26 0.52 0.4 0.23
1 0.86 0.27 0.2 0.36 0.57 0.38 0.28
TOFU−5 TOFU−4 SNPC−4 PRDE−1
AK045 AK004 AK041 AK013 AK010 AK009 AK006 AK005
PRDE−1 SNPC−4 TOFU−4 TOFU−5
AK005 AK006 AK009 AK010 AK013 AK041 AK004 AK045
Correlation within 2nd piRNA cluster at 1kb bins
Pearson Correlation
−1.0 −0.5 0.0 0.5 1.0
1
1 0.8
1 0.79 0.86
1 0.74 0.69 0.75
1 0.68 0.75 0.8 0.77
1 0.91 0.67 0.79 0.84 0.82
1 0.76 0.69 0.52 0.76 0.74 0.63
1 0.95 0.75 0.68 0.51 0.75 0.69 0.62
TOFU−5 TOFU−4 SNPC−4 PRDE−1
AK045 AK004 AK041 AK013 AK010 AK009 AK006 AK005
PRDE−1 SNPC−4 TOFU−4 TOFU−5
AK005 AK006 AK009 AK010 AK013 AK041 AK004 AK045

# Figure S4

A

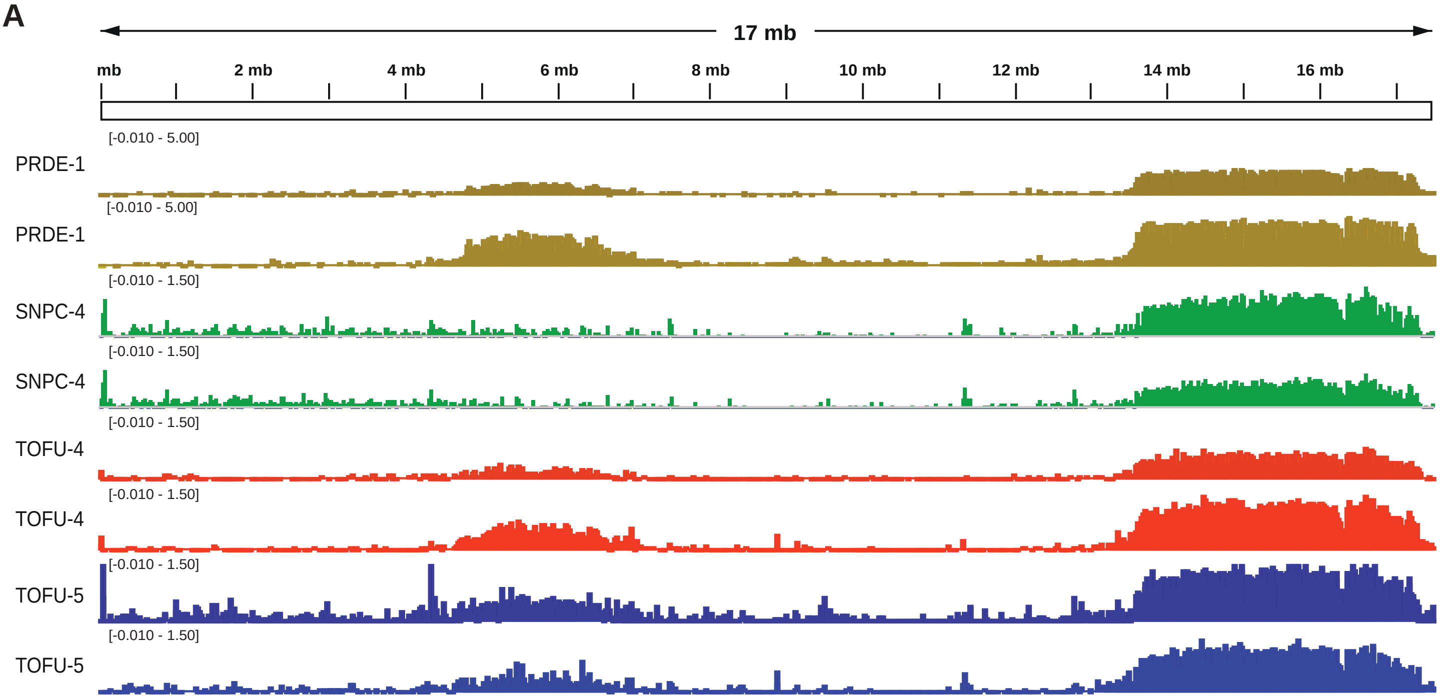
17 mb
mb
2 mb
4 mb
6 mb
8 mb
10 mb
12 mb
14 mb
16 mb
[-0.010 - 5.00]
PRDE-1
[-0.010 - 5.00]
PRDE-1
[-0.010 - 1.50]
SNPC-4
[-0.010 - 1.50]
SNPC-4
[-0.010 - 1.50]
TOFU-4
[-0.010 - 1.50]
TOFU-4
[-0.010 - 1.50]
TOFU-5
[-0.010 - 1.50]
TOFU-5

B

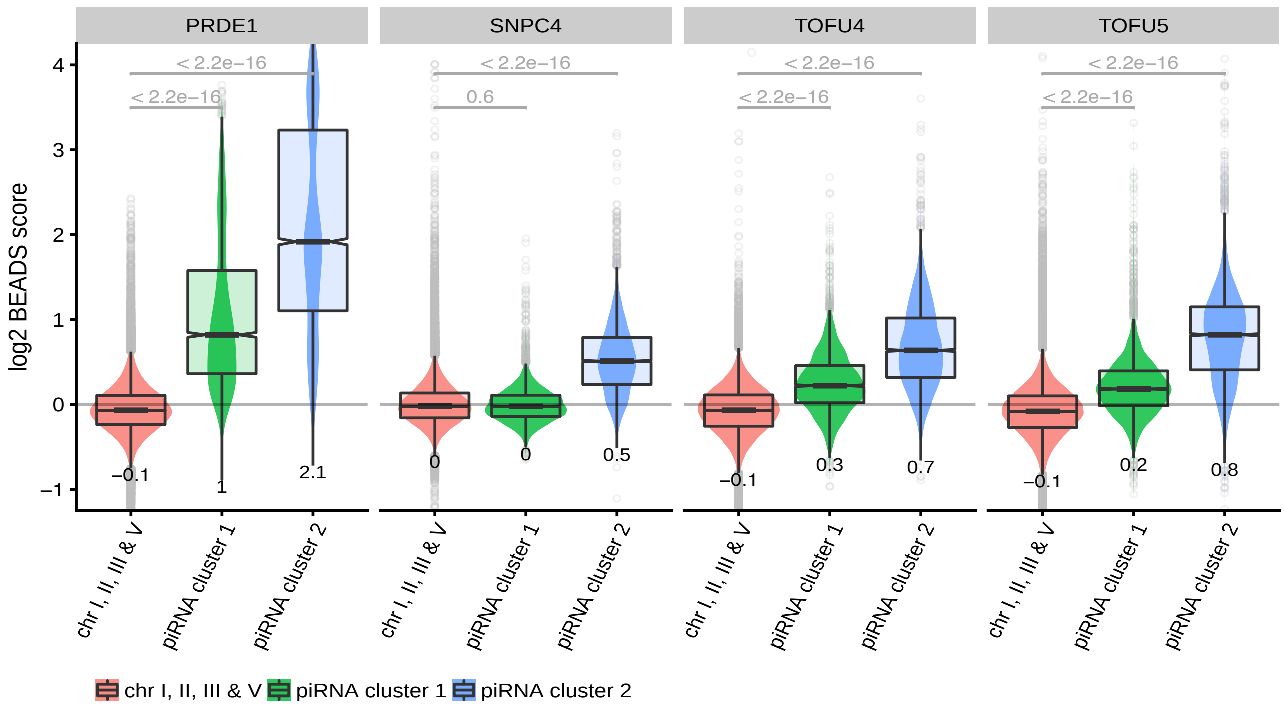
PRDE1
SNPC4
TOFU4
TOFU5
log2 BEADS score
< 2.2e−16
< 2.2e−16
0.6
−0.1
1
2.1
0
0
0.5
0.3
0.7
0.2
0.8
chr I, II, III & V
piRNA cluster 1
piRNA cluster 2
chr I, II, III & V
piRNA cluster 1
piRNA cluster 2

# Figure S5

A

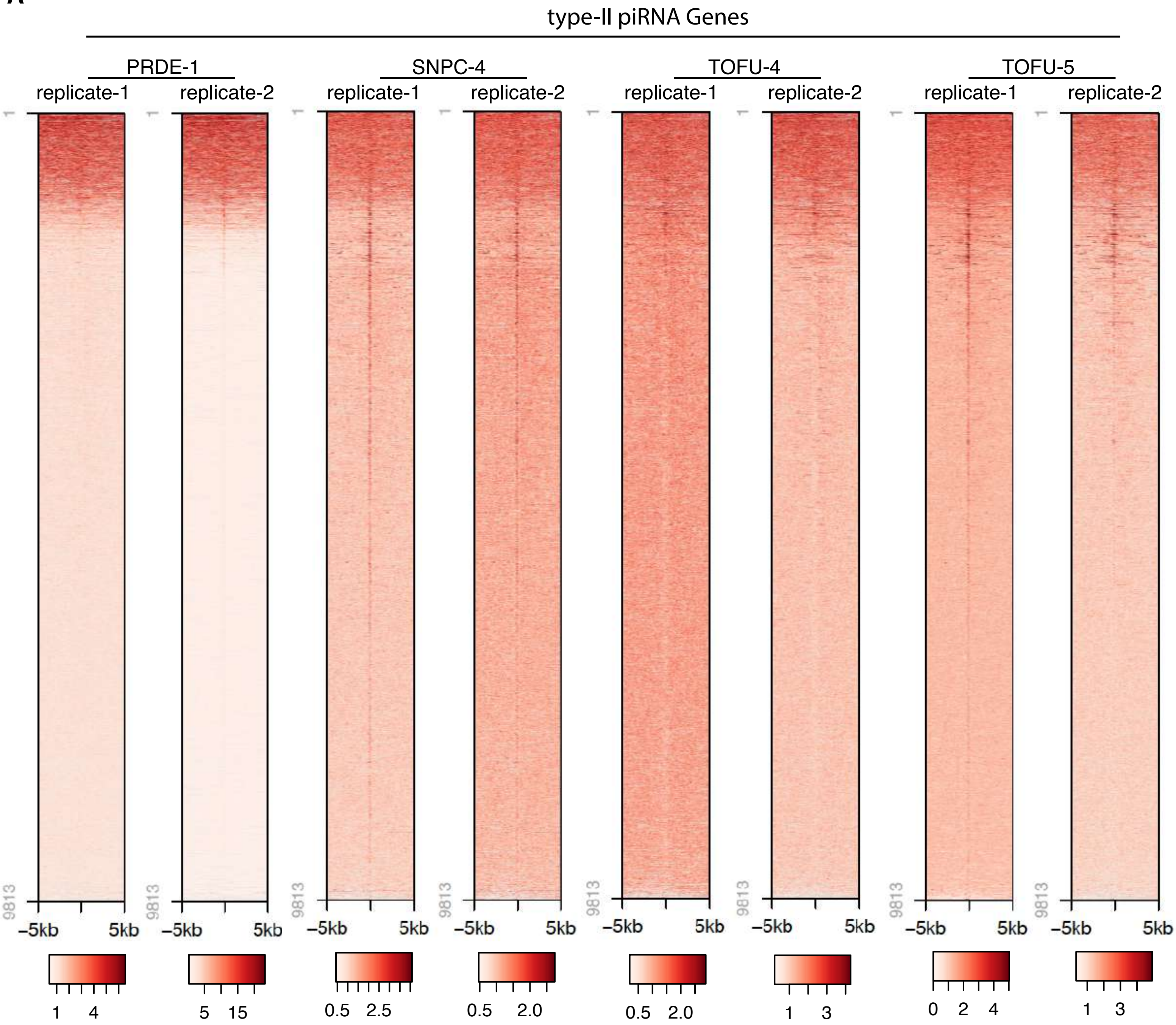
type-II piRNA Genes
PRDE-1
SNPC-4
TOFU-4
TOFU-5
replicate-1
replicate-2
replicate-1
replicate-2
replicate-1
replicate-2
replicate-1
replicate-2
1
9813
−5kb
5kb
1 4
5 15
0.5 2.5
0.5 2.0
0.5 2.0
1 3
0 2 4
1 3

B

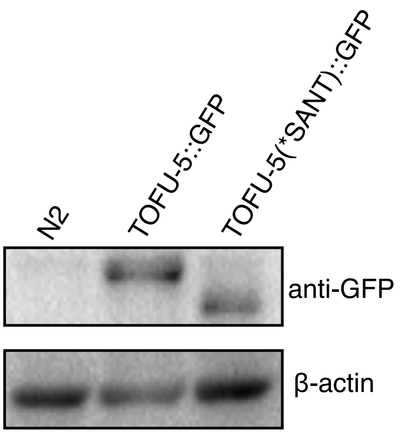
N2
TOFU-5::GFP
TOFU-5(*SANT)::GFP
anti-GFP
β-actin

**Figure S6**

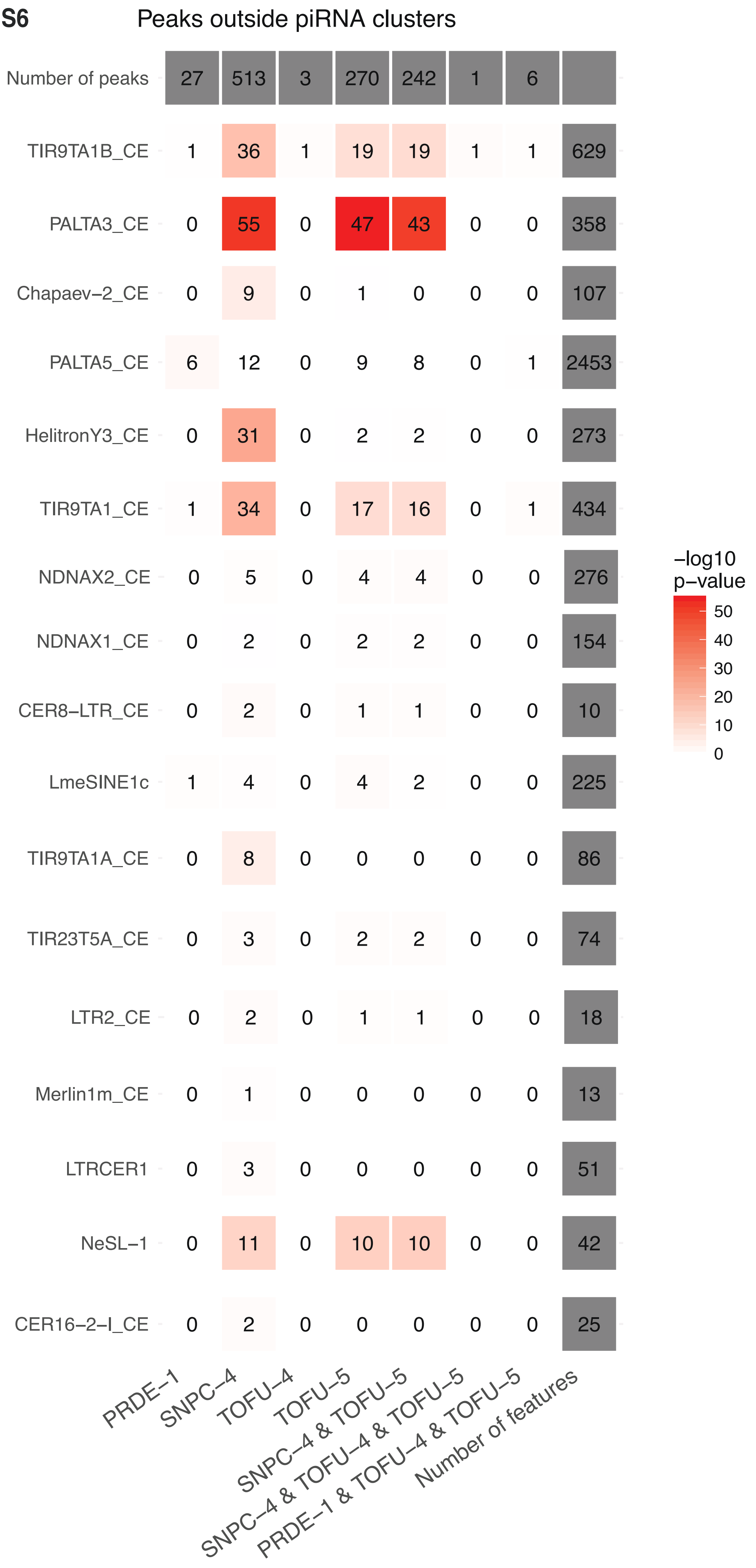
Peaks outside piRNA clusters
Number of peaks 27 513 3 270 242 1 6
TIR9TA1B_CE 1 36 1 19 19 1 1 629
PALTA3_CE 0 55 0 47 43 0 0 358
Chapaev−2_CE 0 9 0 1 0 0 0 107
PALTA5_CE 6 12 0 9 8 0 1 2453
HelitronY3_CE 0 31 0 2 2 0 0 273
TIR9TA1_CE 1 34 0 17 16 0 1 434
NDNAX2_CE 0 5 0 4 4 0 0 276
NDNAX1_CE 0 2 0 2 2 0 0 154
CER8−LTR_CE 0 2 0 1 1 0 0 10
LmeSINE1c 1 4 0 4 2 0 0 225
TIR9TA1A_CE 0 8 0 0 0 0 0 86
TIR23T5A_CE 0 3 0 2 2 0 0 74
LTR2_CE 0 2 0 1 1 0 0 18
Merlin1m_CE 0 1 0 0 0 0 0 13
LTRCER1 0 3 0 0 0 0 0 51
NeSL−1 0 11 0 10 10 0 0 42
CER16−2−I_CE 0 2 0 0 0 0 0 25
PRDE−1
SNPC−4
TOFU−4
TOFU−5
SNPC−4 & TOFU−5
SNPC−4 & TOFU−4 & TOFU−5
PRDE−1 & TOFU−4 & TOFU−5
Number of features
−log10
p−value
50
40
30
20
10
0

## Figure S7

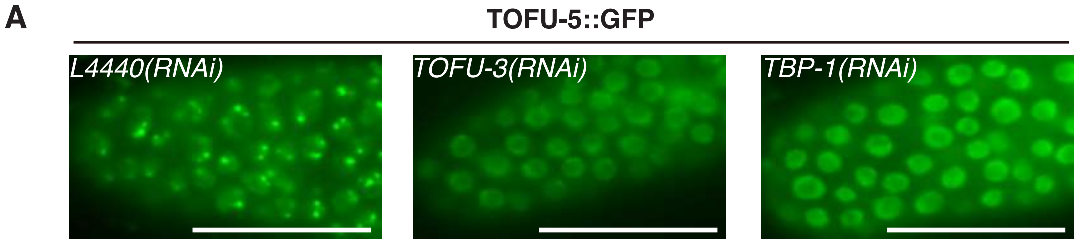
A
TOFU-5::GFP
L4440(RNAi)
TOFU-3(RNAi)
TBP-1(RNAi)

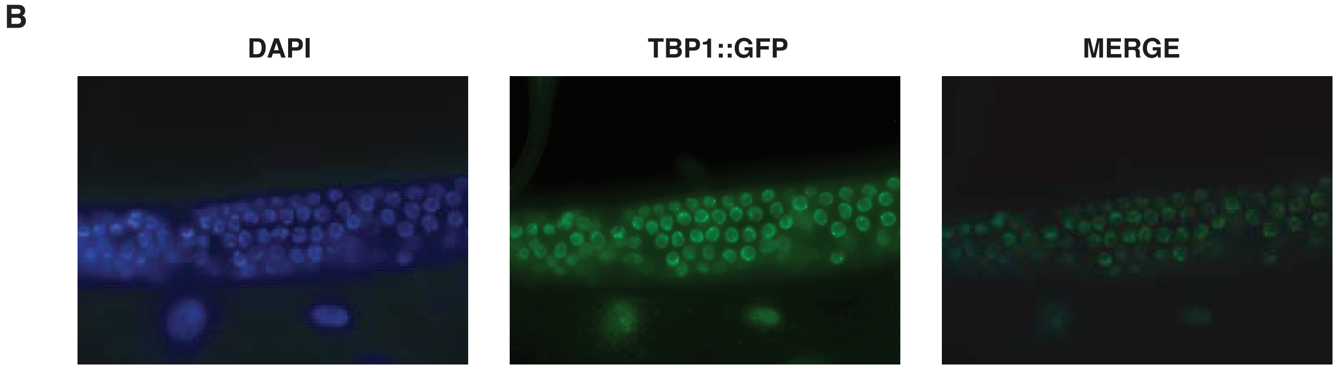
B
DAPI
TBP1::GFP
MERGE
